# Enhanced cerebral oxygenation during mental and physical activity in older adults is unaltered by amnestic mild cognitive impairment 

**DOI:** 10.3389/fphys.2025.1535045

**Published:** 2025-05-12

**Authors:** Stephanie Elbanna, Christopher Cortez, Elaina Smith, Jewelia Rattanavong, Sarah Ross, Geoffrey Kline, April Wiechmann, Hannah Dyson, Robert T. Mallet, Xiangrong Shi

**Affiliations:** ^1^ Department of Pharmacology and Neuroscience, University of North Texas Health Science Center, Fort Worth, TX, United States; ^2^ Department of Internal Medicine, University of North Texas Health Science Center, Fort Worth, TX, United States; ^3^ Department of Physiology and Anatomy, University of North Texas Health Science Center, Fort Worth, TX, United States

**Keywords:** cerebral tissue oxygenation, heart rate response, isometric handgrip, neurovascular coupling, oxyhemoglobin, serial sevens test

## Abstract

**Background:**

The impact of amnestic mild cognitive impairment (aMCI) on cerebral oxygenation and cardiovascular responses to mental and physical challenges in elderly adults is unclear. This study compared the responses to mental (serial sevens test) and physical (isometric handgrip) challenges in older adults with vs. without aMCI.

**Methods:**

Thirty-one aMCI (71.5 ± 1.1 years old) and 30 cognitively normal (70.8 ± 1.1 years old) adults participated in the study. Heart rate (HR), mean arterial pressure (MAP), systemic arterial oxygen saturation (SaO_2_), prefrontal cortical oxyhemoglobin (O_2_Hb) and deoxyhemoglobin (HHb) contents, and tissue oxygen saturation (ScO_2_) were continuously monitored during 2-min serial sevens mental arithmetic test and 1-min isometric handgrip at 30% of maximal voluntary contraction. Test results in the aMCI vs. non-MCI subjects were compared by two-factor ANOVA.

**Results:**

Cardiovascular and tissue oxygenation responses to testing were similar in the two groups. Although MAP increased similarly during the mental and physical challenges, increases in HR (P = 0.020), SaO_2_ (P < 0.001), ScO_2_ (P = 0.001) and O_2_Hb (P = 0.022) were greater during the mental vs. physical challenges in both aMCI and cognitively normal subjects.

**Conclusion:**

The mental arithmetic challenge increased the metabolic demand of the prefrontal cortex to a greater extent than the physical task. Cerebral O_2_ content increased more appreciably during the mental vs. physical challenges, in parallel with greater increases in HR. However, aMCI did not alter these physiological responses to mental or physical challenges.

## Introduction

The impacts of cognitive impairment on cerebrovascular function and cerebral O_2_ content during mental and physical challenges are unclear. Cardiovascular responses to face-name recall challenges were dampened in older adults with mild cognitive impairment (MCI) vs. younger, cognitively normal adults (Henley et al., 2018). Paralleling the attenuated cardiovascular response, cerebral blood flow, assessed by arterial spin-labeling magnetic resonance imaging (MRI) also decreased in MCI vs. cognitively intact subjects, although the MRI measurements of cerebral blood flow were obtainable only after the mental challenges (Henley et al., 2018). Near-infrared spectroscopy (NIRS) monitoring of cerebral tissue oxygenation (ScO_2_) and oxyhemoglobin (O_2_Hb) is less movement-sensitive than MRI. Thus, NIRS is frequently applied to monitor cerebrovascular perfusion and cerebrocortical oxygenation during mental challenges in older or fragile subjects with Alzheimer’s disease (Herrmann et al., 2008), dementia (Takahashi et al., 2015) and MCI (Takahashi et al., 2022). Herrmann et al. (2008) reported blunting of cerebral O_2_Hb responses to verbal fluency tests in Alzheimer’s patients, but Takahashi et al. (2015) detected no change in NIRS-monitored prefrontal cortical blood flow in dementia patients. Prefrontal cortical O_2_Hb increased similarly during verbal fluency tests in healthy younger and older adults, while O_2_Hb increased to a greater extent in the older subjects during finger tapping physical tests (Takahashi et al., 2022). Furthermore, brief (15 s) verbal fluency or finger tapping tests elicited similar O_2_Hb responses in older adults with or without MCI (Takahashi et al., 2022). The question remained if MCI altered cardiovascular and cerebrocortical oxygenation responses to more protracted mental and physical challenges in older adults.

Cerebral perfusion and tissue oxygenation increase to meet the increased metabolic demands of the brain imposed by mental or physical activity (Secher et al., 2008). Mental and physical challenges engage sympathetic nervous activity to increase cardiac output and arterial pressure (Wasmund et al., 2002), albeit by distinct physiological mechanisms. Mental activity elicits central command responses in the brain, while the pressor reflex (McCloskey and Mitchell, 1972) activated by input from contracting muscle is the predominant response to physical exercise (Kaufman et al., 1983). In studies comparing cardiovascular responses to mental vs. physical activity in young adults, Rousselle et al. (1995) reported greater tachycardic responses to mental arithmetic tasks vs. cycle ergometer exercise at 50W, although cycling provoked greater increases in whole body oxygen consumption and ventilation. In contrast, Wasmund et al. (2002) reported greater increases in heart rate and mean arterial pressure during 5-min static handgrip at 25%–30% of maximal voluntary contraction (MVC) vs. 5-min mental arithmetic, also in young adults. However, tachycardic responses to mental (Reddy et al., 2024) and physical (Boutcher and Stocker, 1999) challenges are diminished in healthy older adults. Available data on cardiovascular responses to mental and physical challenges in elderly adults with or without MCI remain inconclusive.

This study tested the hypotheses that (1) cerebral O_2_ saturation increases during mental but not physical challenges in older adults, and (2) amnestic MCI (aMCI) attenuates the increase in cerebrocortical oxygenation during mental challenges, possibly due to less effective neurovascular coupling. The responses of heart rate, arterial pressure and cerebrocortical oxygenation to mental challenge and isometric handgrip were compared in elderly adults with vs. without aMCI. Since 15-s finger tapping elicited no difference in cerebral O_2_Hb in the older subjects with MCI vs. unimpaired cognition, but aroused a greater response in older than younger subjects likely due to increasing physical demand with age (Takahashi et al., 2022), we employed 1 min isometric handgrip without limb movement as the physical challenge. This protocol was expected to elicit a robust cardiovascular response with minimal disturbance of overall homeostasis in elderly subjects.

## Materials and methods

### Study participants

Thirty-one aMCI (23 women) and 30 cognitively normal (27 women) older adults signed the informed consent prior to study enrollment. The study protocol and consent form were reviewed and approved by the North Texas Regional Institutional Review Board for Protection of Human Subjects. All subjects passed a physical screening prior to experimental testing. Inclusion criteria included ability to visit the lab for the experiments, free of clinical depression at the time of enrollment, post-menopausal if female, and ≥6 months stabilization of chronic conditions including hypertension, coronary artery disease, diabetes or metabolic disease, chronic bronchitis, degenerative osteoporosis/arthritis and/or other aging-related chronic conditions. Exclusion criteria were a diagnosis of AD-dementia, impaired independent daily functioning, mini-mental state exam (MMSE) score <20 and/or clinical dementia rating (CDR) ≥1; unable to visit the lab independently; active smoker; expecting major surgery or transplant; having uncontrolled chronic conditions including systolic-diastolic pressures over 150/90 mmHg with medications, diabetes, chronic renal failure, recurrent chest pain, seizures or epilepsies, brain aneurysm, uncontrolled allergic rhinitis, cancer, infectious disease, regular premature ventricular contractions, myocardial ischemia or infarct, severe head injury or traumatic brain injury, stroke, currently diagnosed depression, or having metallic implants either above the neck or active in nature (e.g., cardiac pacemaker, stimulators, infusion pumps). Diagnosis of aMCI was based on in-person evaluation of clinical dementia rating (CDR) and a battery of neurocognitive function tests including verbal-memory and visuospatial-memory conducted by a geriatric neuropsychologist or psychiatrist, according to the consensus criteria (Winblad et al., 2004; Petersen et al., 2010). The aMCI subjects had a self- or family member-reported memory complaint, a clinical dementia rating ≤0.5, and at least one memory testing score ≥1 standard deviation below the age- and education-adjusted normal group mean, but had normal daily living functionality and were not demented (Smith et al., 2025). Table 1 summarizes the physical and cognitive characteristics of the subjects.

**TABLE 1:** Physical and cognitive characteristics.

	aMCI n = 31	Normal n = 30	P Value
Man vs. Woman	8:23	3:27	0.182
Age (year)	71.5 ± 1.1	70.8 ± 1.1	0.661
Weight (kg)	71.4 ± 3.0	75.4 ± 2.5	0.307
Height (m)	1.66 ± 0.02	1.65 ± 0.01	0.528
Education (year)	16.1 ± 0.2	15.9 ± 0.3	0.616
Geriatric Depression Scale (score)	1.0 ± 0.2	1.1 ± 0.2	0.861
Clinical Dementia Rating (score)	0.5 ± 0.0	0.3 ± 0.1	0.002
Mini-Mental State Examination (score)	27.8 ± 0.2	28.7 ± 0.2	0.014
Trail Making Test –A (sec)	44.6 ± 3.0	28.8 ± 1.3	<0.001
Verbal Memory (score)	5.8 ± 0.4	8.8 ± 0.2	<0.001
Visuospatial memory (score)	5.0 ± 0.6	8.7 ± 0.3	<0.001
Number (%) of subjects taking prescribed medications			
Hypertension	17 (54.8)	12 (40.0)	0.309
Hyper-cholesterol/Hyper-lipidemia	11 (35.5)	10 (33.3)	1.000
Hyperglycemia	7 (22.6)	3 (10.0)	0.301
Anxiety/Depression	11 (35.5)	7 (23.3)	0.402
Hypothyroid	6 (19.4)	9 (30.0)	0.384
Reflux/Gastric acid	7 (22.6)	8 (26.7)	0.772
Allergy	8 (25.8)	7 (23.3)	1.000
Sleep aid	6 (19.4)	3 (10.0)	0.473
Hormone replacement	5 (16.6)	3 (10.0)	0.707
Arthritis	4 (12.9)	3 (10.0)	1.000
Asthma	5 (16.6)	2 (6.7)	0.425

The scores of verbal memory and visuospatial memory are 10-min delayed-recall in words and 30-min delayed-recall in location of line-sketches assessed with California-Verbal-Learning Memory – 2nd edition Short-Form and Brief-Visuospatial-Memory-Test–Revised, respectively.

### Measurements

Cardiovascular and cerebral tissue oxygenation responses to mental and physical challenges were assessed during 2-min serial sevens test and 1-min isometric handgrip at 30% of individual MVC, respectively. Handgrip force was measured with a digital hand dynamometer (Smedley Digital Hand Dynamometer Model 12–0286, Baseline® Evaluation Instruments, Elmsford, NY, United States). Before the experiment, subjects visited the laboratory for orientation to the testing procedures and methods of measurement. During orientation, maximal voluntary contraction force (MVC) of the subject’s dominant hand was taken as the strongest handgrip of three trials.

**FIGURE 1:**
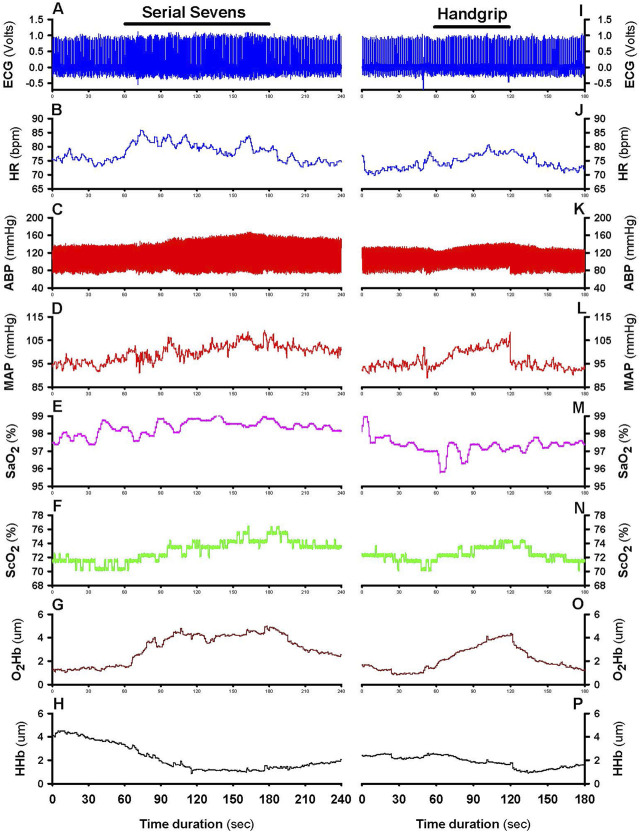
Typical recording of cardiovascular and cerebrocortical oxygenation variables. Continual recordings from a 76-year-old female subject before, during and after 2-min mental challenge (left: panels **(A–H)**) and 1-min physical challenge (right: panels **(I–P)**). After pre-test baseline (0–60 s), the mental challenge was applied at 60–180 s, and the physical challenge at 60–120 s, indicated by horizontal bars in panels **(A,I)**, followed by recovery. Panels from top to bottom show a standard lead II electrocardiograph (ECG); heart rate (HR) derived from analog ECG; beat-to-beat arterial blood pressure (ABP); mean arterial pressure (MAP) derived from analog ABP; arterial oxygen saturation (SaO_2_); cerebral tissue oxygen saturation (ScO_2_); cerebral tissue oxyhemoglobin (O_2_Hb); and deoxyhemoglobin (HHb).

Ambient temperature and relative humidity were maintained at 24°C ± 1°C and 50%–58%, respectively, throughout the experiments. Heart rate (HR) was continuously monitored by standard lead II electrocardiography (BIOPAC Model ECG100C, Santa Barbara, CA). Beat-to-beat systolic (SP) and diastolic (DP) arterial pressures were monitored by double finger cuffs placed on the proximal phalanges of the index and middle fingers of the non-dominant hand (CNAP 500, Graz, Austria). Before each use the finger cuff sensors were calibrated against blood pressures measured with a standard brachial arterial cuff. Mean arterial pressure (MAP) was taken as 1/3 of systolic pressure plus 2/3 of diastolic pressure. Systemic arterial O_2_ saturation (SaO_2_) was monitored in the right earlobe by a transcutaneous sensor (TOSCA 500, Radiometer America Inc., Westlake, OH, United States) maintained at 42°C to dilate and thereby arterialize the cutaneous capillary blood in the earlobe. Cerebral tissue O_2_ saturation (ScO_2_) and oxygenated (O_2_Hb) and deoxygenated hemoglobin (HHb) of the prefrontal cortex were monitored by NIRS (NIRO-200, Hamamatsu Photonics, Bridgewater, NJ, United States) with a sensor placed on the right forehead at an analog signal output of 1 Hz. A previous study showed no difference in NIRS O_2_Hb measurements between the left and right prefrontal cortical lobes in subjects with or without MCI (Takahashi et al., 2022). Baseline ScO_2_, O_2_Hb and HHb were reset before initializing the measurement. All analog data were continuously digitized on-line at 250 Hz by a computer interfaced with a data acquisition system (MP150 BIOPAC, Santa Barbara, CA). Figure 1 presents a typical recording of the monitored variables.

### Study protocol

After collection of ∼2 min baseline data, both serial sevens and handgrip tests were conducted with the subject in the supine position with minimum body movement. In the authors’ experience, cerebral tissue oxygenation and hemodynamic variables in older subjects are more stable in the supine position. Before the serial sevens test, the subject was instructed to count down by intervals of seven from a random starting number (i.e., 100, 99 or 98) as quickly and accurately as possible. If a mistake was made, the correct number was provided, and the subject continued the countdown from the correct number. Handgrip test with the dominant hand was performed after ∼5 min recovery from serial sevens test. The subject was directed to maintain a handgrip approximating 30% of the subject’s MVC with direct visual feedback and verbal encouragement by a research assistant. A handgrip strength of 30% MVC was utilized, which the elderly subjects were able to maintain for at least 1 minute without reporting discomfort. HR, SP, DP, SaO_2_, ScO_2_, O_2_Hb and HHb were continuously monitored during serial sevens test and 1-min isometric handgrip (Figure 1).

### Data analysis

A ∼30 s segment of continuous, stable analog data collected before the challenges was averaged to obtain baseline values. The differences between baseline values and mean values of ∼30-s recordings during the 1st and 2nd min of serial sevens test and 1-min isometric handgrip were taken as the responses to mental and physical challenges, respectively. Two-factor analyses of variance (ANOVA) were conducted to assess the statistical significance of the differences of the baseline values and the responses to the challenges between MCI and non-MCI subjects (group factor) during mental vs. physical challenges (test factor). In addition, two-factor ANOVA was conducted to examine the response differences in two groups (group factor) between the 1st and 2nd minute during mental challenge (minute factor). Proportions of the subject’s distributions in the aMCI and normal groups were compared using Fisher’s exact test for two-tailed probability. Differences in numerical variables of basic characteristics between the groups were tested using t-test for two independent groups. Statistical analyses were performed with Statistical Analysis System software (SAS Version 9.4, Cary, NC, United States). Values are reported as group mean ± standard error of the mean (SEM). Statistical significance was accepted at P ≤ 0.05.

## Results

There was no significant difference in age or education attainment between the groups (Table 1). Both groups had a normal Geriatric Depression Scale score (below 5 out of 15 points). Mini-mental state examination score was slightly but significantly lower in the aMCI than normal subjects. Moreover, both verbal and visuospatial memory scores were significantly lower in the aMCI than normal groups. Most of the elderly subjects, 30 of 31 in the aMCI group and 26 of 30 in the normal group took prescribed medications daily. Sixteen subjects in the aMCI and normal groups, respectively, took more than 2 medications daily. The percentages of subjects who took the medications were statistically similar in the two groups (Table 1).

### Baseline variables

Table 2 summarizes baseline values for the subjects with and without aMCI prior to serial sevens and isometric handgrip tests. None of the pretest baseline values differed in the aMCI vs. control subjects. Resting SP (P = 0.043) and O_2_Hb (P = 0.017) were significantly higher in the aMCI vs. non-MCI groups at baseline. However, *post hoc* analysis indicated that between-group differences in baseline SP and O_2_Hb did not attain statistical significance before mental (SP: P = 0.175; O_2_Hb: P = 0.096) or physical (SP: P = 0.138; O_2_Hb: P = 0.089) challenges. Serial sevens test performance did not differ in terms of the number to count down (aMCI: 18.7 ± 1.4; non-MCI: 20.3 ± 1.6; P = 0.467) or mistakes made (aMCI: 3.5 ± 0.4; non-MCI: 2.9 ± 0.4; P = 0.346). During handgrip the maximum isometric force of voluntary contraction (MVC) and 30% of MVC were similar (P = 0.746) in aMCI (22.5 ± 1.2 or 6.75 ± 0.36 kg) and non-MCI (21.9 ± 1.6 or 6.56 ± 0.48 kg) subjects.

**TABLE 2:** Baseline variables prior to mental (serial sevens test) and physical (isometric handgrip) challenges.

	Mental challenge		Physical challenge		P Values	
	aMCI (n = 31)	Normal (n = 30)	aMCI (n = 31)	Normal (n = 30)	Group	Test
HR (bpm)	69 ± 2	68 ± 2	68 ± 2	67 ± 2	0.432	0.736
SP (mmHg)	138 ± 2	134 ± 4	136 ± 2	132 ± 2	0.043	0.442
DP (mmHg)	75 ± 2	76 ± 2	74 ± 2	74 ± 2	0.773	0.609
MAP (mmHg)	96 ± 2	95 ± 2	95 ± 2	94 ± 2	0.483	0.478
SaO_2_ (%)	97.1 ± 0.2	96.4 ± 0.2	96.8 ± 0.3	96.6 ± 0.3	0.075	0.997
ScO_2_ (%)	65.1 ± 0.8	66.5 ± 1.1	65.1 ± 0.9	66.9 ± 1.0	0.094	0.846
O_2_Hb (µm)	7.72 ± 0.66	6.08 ± 0.71	7.69 ± 0.64	6.06 ± 0.69	0.017	0.972
HHb (µm)	1.33 ± 0.20	1.48 ± 0.17	1.78 ± 0.34	1.36 ± 0.18	0.556	0.476

Baseline variables before 2-min serial sevens test (mental challenge) and 1-min isometric handgrip at 30% of the maximal voluntary contraction (physical challenge) for group subjects with amnestic mild cognitive impairment (aMCI) and normal cognition (Normal). HR: heart rate; SP: systolic blood pressure; DP: diastolic blood pressure; MAP: mean arterial pressure; SaO_2_: systemic arterial oxygen saturation; ScO_2_: cerebral tissue oxygenation; O_2_Hb: cerebral oxygenated hemoglobin; HHb: cerebral de-oxygenated hemoglobin. P values are the outcomes of two-factor ANOVA Data represent group mean ± standard error of the mean (SEM). For O_2_Hb and HHb variables n = 30 in aMCI and n = 29 in Normal groups.

### Cardiovascular responses to mental and physical challenges

**FIGURE 2:**
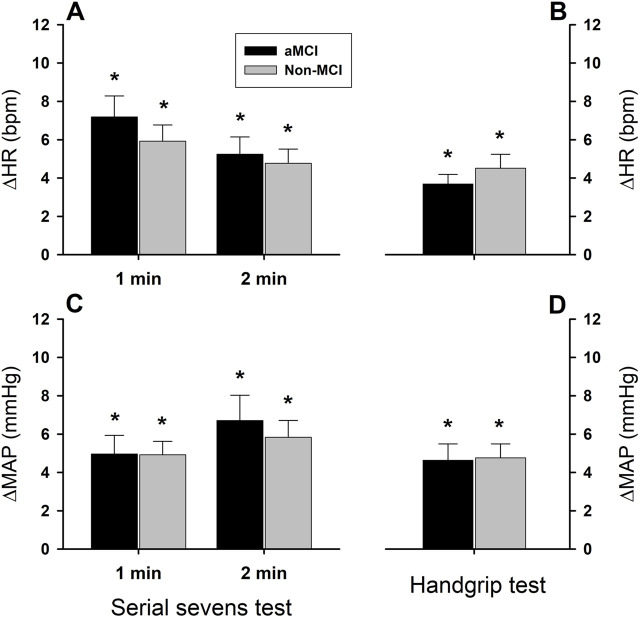
Heart rate and mean arterial pressure responses to mental and physical challenges. Both heart rate (panels **(A,B)**) and mean arterial pressure (panels **(C,D)**) increased (*: P < 0.05) during 2-min serial sevens test and 1-min isometric handgrip at 30% of the subject’s maximal voluntary contraction. There were no statistically significant effects of aMCI on the changes in heart rate (ΔHR) or mean arterial pressure (ΔMAP) (group factor P = 0.975 for ΔHR, P = 0.840 for ΔMAP). The serial sevens test provoked greater HR increases than isometric handgrip (test factor P = 0.020), but the tests produced similar increases in MAP (test factor P = 0.260). Responses in HR and MAP were not significantly different between the 1st and 2nd min during mental challenge (minute factor P = 0.091 for ΔHR and P = 0.185 for ΔMAP). Data were averaged over ∼30 s continuous analog recordings encompassing the peak HR and MAP responses. Values are group means ± standard error of the mean (SEM).

During serial sevens and isometric handgrip tests, both HR and MAP increased significantly in the aMCI and non-MCI subjects (Figure 2). These cardiovascular responses did not differ between the groups (group factor P = 0.975 for HR and P = 0.839 for MAP). The serial sevens test produced a greater increase in HR (ΔHR) than isometric handgrip (test factor P = 0.020). ∆HR (beats•min^−1^) equaled 7.2 ± 1.1 and 5.2 ± 0.9 in the aMCI group and 5.9 ± 0.9 and 4.8 ± 0.7 in the non-MCI group during the 1st and 2nd min of the serial sevens test, respectively, vs. 3.7 ± 0.5 in the aMCI group and 4.5 ± 0.7 in the non-MCI group during 1 min isometric handgrip (Figures 2A,B). ΔHR tended to be greater in the first minute of the mental challenge (minute factor P = 0.091). However, MAP increased to similar extents during the different challenges (test factor P = 0.265). ∆MAP (mmHg) equaled 5.0 ± 1.0 and 6.7 ± 1.3 in the aMCI group and 4.9 ± 0.7 and 5.8 ± 0.9 in the non-MCI group during the 1st and 2nd min serial sevens test, respectively, vs. 4.5 ± 0.7 in the aMCI group and 4.6 ± 0.9 in the non-MCI group during 1-min isometric handgrip (Figures 2C,D). ∆MAP did not differ between the 1st and 2nd minute of the serial sevens test (minute factor P = 0.185).

### Impact of mental and physical challenges on arterial and cerebral tissue oxygenation

**FIGURE 3:**
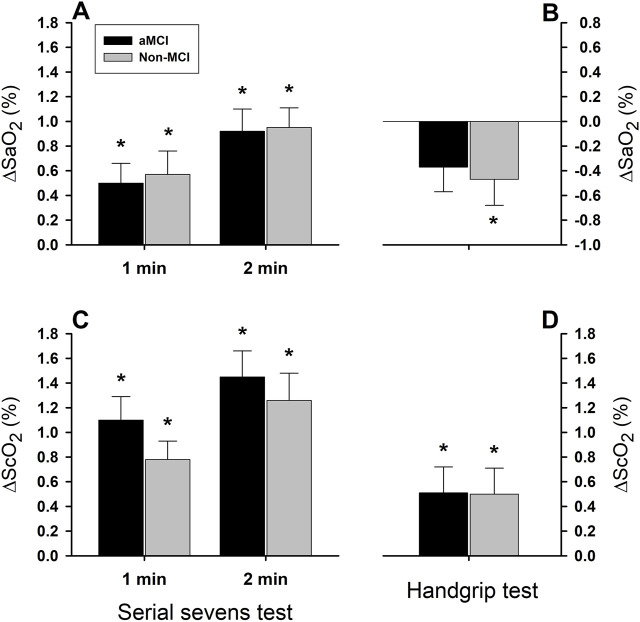
Systemic arterial and cerebral tissue oxygen saturation responses to mental and physical challenges. Arterial oxygen saturation (SaO_2_) significantly increased (*: P < 0.05) during serial sevens mental challenge in both aMCI and non-MCI groups (Panel **(A)**), but decreased during physical challenge with isometric handgrip (Panel **(B)**). Although two-factor ANOVA showed the test factor to be statistically significant (P < 0.001), the group factor was not statistically significant (P = 0.881) for the SaO_2_ response. Cerebral tissue oxygenation (ScO_2_) increased during serial sevens test (Panel **(C)**) and during isometric handgrip (Panel **(D)**) in both groups. Responses in ScO_2_ were significantly greater during serial sevens test than during isometric handgrip (test factor P = 0.001). However, these responses were not different between two groups (group factor P = 0.444). Both SaO_2_ and ScO_2_ responses were augmented during the 2nd vs. 1st min serial sevens test (minute factor P = 0.023 for SaO_2_ and P = 0.037 for ScO_2_). Values are group means ± SEM. Data were averaged over ∼30 s continuous analog recordings encompassing the peak HR and MAP responses.

Arterial oxygen saturation (Figure 3A) increased significantly in both the aMCI and non-MCI groups during the 1st min (ΔSaO_2_ = 0.50 ± 0.16% in the aMCI group and 0.57% ± 0.19% in the non-MCI group) and 2nd min mental challenge (ΔSaO_2_ = 0.92 ± 0.18% in the aMCI group and 0.95% ± 0.16% in the non-MCI group). The magnitude of ΔSaO_2_ was greater during the 2nd min than the 1st min (minute factor P < 0.001). In contrast, ∆SaO_2_ during the 1-min physical challenge tended to decrease from baseline in the aMCI (−0.37% ± 0.20%, P = 0.074) and non-MCI (−0.47% ± 0.21%, P = 0.039) groups (Figure 3B). Although ∆SaO_2_ was greater during the mental vs. physical challenges (test factor P < 0.001), it did not differ in the aMCI vs. non-MCI subjects (group factor P = 0.881).

Cerebral tissue oxygenation (ScO_2_) significantly increased during the 1st and 2nd min mental challenge (Figure 3C) and 1-min physical challenge (Figure 3D) in both groups. However, the magnitude of ∆ScO_2_ was significantly greater during mental vs. physical challenges (test factor P = 0.001), and was greater during the 2nd than the 1st min of the mental challenge (minute factor P = 0.037). The ∆ScO_2_ responses did not differ significantly (group factor P = 0.488) in the aMCI vs. non-MCI subjects during the 1st (1.10% ± 0.19% vs. 0.78% ± 0.15%) and 2nd min mental challenge (1.45% ± 0.21% vs. 1.26% ± 0.22%), or during the 1-min physical challenge (0.51% ± 0.21% vs. 0.50% ± 0.21%).

**FIGURE 4:**
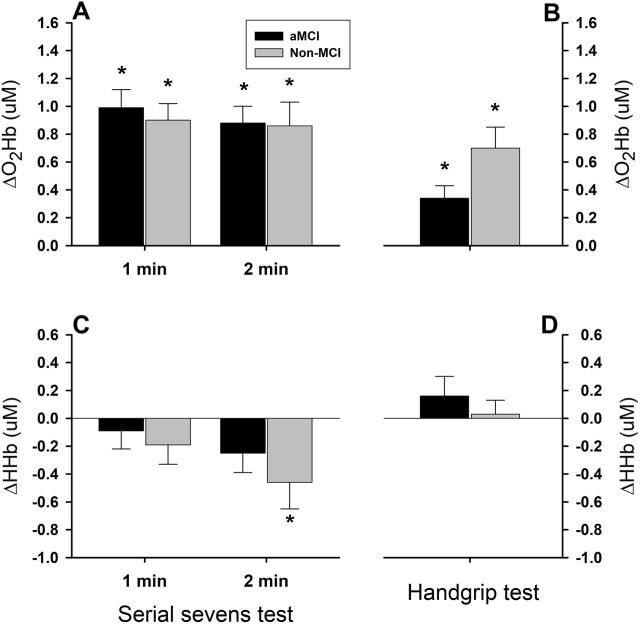
Cerebral tissue oxygenated and deoxygenated hemoglobin responses to mental and physical challenges. Cerebral tissue oxyhemoglobin (O_2_Hb) concentration increased significantly during 2-min serial sevens test (Panel **(A)**) and 1-min isometric handgrip at 30% of the maximal voluntary contraction (Panel **(B)**), while deoxyhemoglobin (HHb) concentration did not change, except the responses during the 2nd min serial sevens test which tended to be decreased (Panels **(C,D)**). Oxyhemoglobin responses did not differ in the aMCI vs. non-MCI subjects (group factor P = 0.178). Increases in oxyhemoglobin (ΔO_2_Hb) were significantly greater during serial sevens test than during isometric handgrip (test factor P = 0.001). The responses of cerebral tissue deoxyhemoglobin during the mental and physical challenges showed a directional difference (test factor P = 0.001). However, HHb responses in the two groups did not differ (group factor P = 0.238). Furthermore, the HHb responses during the 1st and 2nd min serial sevens test did not differ (minute factor P = 0.152), despite the trend toward decreased HHb in the subjects with aMCI (−0.25 ± 0.14 µm, P = 0.075) and normal cognition (−0.46 ± 0.19 µm, P = 0.021) during the 2nd min serial sevens test. Data (group mean ± SEM) were averaged over ∼30 s continuous analog recordings encompassing the peak HR and MAP responses.

Oxygenated hemoglobin (O_2_Hb) increased significantly in the aMCI and non-MCI groups during both serial sevens and isometric handgrip tests (Figures 4A,B), albeit to a greater extent (test factor P < 0.001) during the mental challenge (1st min: aMCI: 0.99 ± 0.13 μM, non-MCI: 0.90 ± 0.13 μM; 2nd min: aMCI: 0.88 ± 0.12 μM, non-MCI: 0.86 ± 0.17 μM) than the physical challenge (aMCI: 0.34 ± 0.09 μM; non-MCI: 0.70 ± 0.15 μM). The magnitude of ∆O_2_Hb did not differ in the aMCI vs. non-MCI subjects (group factor P = 0.178). The responses in ∆O_2_Hb were similar between the 1st and 2nd min mental challenge (minute factor P = 0.610). However, ∆HHb did not change significantly in either group during the 1st min mental or the 1-min physical challenges (Figures 4C,D). During the 2nd min serial sevens test, HHb declined in the non-MCI subjects (−0.46 ± 0.19 µm, P = 0.021) and tended to decrease in the aMCI group (−0.25 ± 0.14 µm, P = 0.075). However, although the test factor was significant (two-factor ANOVA: P = 0.005), the group factor was not (P = 0.238).

## Discussion

This study in elderly adults with normal cognition and amnestic mild cognitive impairment demonstrated that an arithmetic mental challenge (serial sevens test) provoked a more robust tachycardic response than physical challenge (isometric handgrip at 30% of individual MVC), although the two tests elicited similar pressor responses. Furthermore, the mental challenge produced greater increases in prefrontal cortical O_2_Hb and ScO_2_ than the physical challenge. However, none of these responses differed significantly in the elderly adults with aMCI vs. those with normal cognition. These findings indicate that mental tasks provoke greater increases in frontal cortical oxygenation than physical tasks. However, aMCI did not blunt the enhancement of cerebrocortical oxygenation during mentation. This result refuted the hypothesis that the cerebrocortical oxygenation response may be diminished in aMCI, especially during mental challenge, due to impaired neurovascular coupling. These data provide neurophysiological support for applications of mental activity training to enhance cerebral perfusion and oxygenation and delay the onset of cognitive impairment or slow its progression to AD and/or vascular dementia in elderly adults, even those with MCI (Aguirre et al., 2010; Coen et al., 2011; Woods et al., 2012).

### Assessment of cerebral tissue oxygenation with NIRS

Near-infrared spectroscopy (NIRS) of the cerebral cortex is based on oxygenation-dependent near infrared light absorption by the chromophores O_2_Hb and HHb and the relative transparency of human brain tissue to near infrared light (Hoshi and Tamura, 1993; Kato et al., 1993; Villringer et al., 1993). NIRS-detected changes in cerebral O_2_Hb correspond closely to those reported by blood oxygenation-sensitive functional magnetic resonance imaging (BOLD fMRI) (Strangman et al., 2002) with high reproducibility and stability during brain activation (Herrmann et al., 2008). Accordingly, NIRS is applied extensively in clinical research to monitor cerebrovascular function and cerebrocortical metabolism (Agbangla et al., 2017; Gramigna et al., 2017). Cerebral tissue oxygenation (ScO_2_) is estimated from the NIRS-detected ratio, O_2_Hb/(O_2_Hb + HHb). Decreased O_2_Hb and ScO_2_ have been demonstrated during orthostatically induced cerebral hypoperfusion in elderly adults (Mehagnoul-Schipper et al., 2000; Formes et al., 2010; Wang et al., 2020), in parallel with decreased cerebral blood flow velocity (Formes et al., 2010).

In this study, the responses of ∆ScO_2_ (Figure 3) and ∆O_2_Hb (Figure 4) were more robust during serial sevens mental challenge than physical challenge with isometric handgrip. Since ∆ScO_2_ and ∆O_2_Hb represent the balance between cerebrovascular O_2_ delivery and cerebral O_2_ consumption, increases in cerebral tissue oxygenation suggest that the augmented O_2_ delivery exceeded the increased O_2_ consumption during the mental and physical challenges, a finding in keeping with the feedforward neurovascular coupling mechanism (Schaeffer and Iadecola, 2021). Although brain metabolism was not measured directly, it was unlikely the physical challenge increased brain metabolism more than the mental challenge. Thus, a greater ∆ScO_2_ and ∆O_2_Hb during the serial sevens test suggested a greater increase in cerebrovascular perfusion during mental vs. physical challenge. However, HHb did not change appreciably during the 1^st^ min mental challenge or 1-min physical challenges, indicating that the enhanced cerebral perfusion (which cleared HHb) and oxygen delivery (which augmented O_2_Hb) likely exceeded any metabolic increase in HHb. This observation was concordant with previous reports showing ∆HHb to be less affected than ∆O_2_Hb by finger flexion-extension (Strangman et al., 2002) and hand grasping (Arun et al., 2020) physical tasks. Nonetheless, HHb declined during the 2nd vs. 1st min mental challenge (two-factor ANOVA: minute factor P = 0.037), suggesting the increased cerebral perfusion cleared HHb gradually, not abruptly (Figure 4).

### Cardiovascular responses to mental and physical challenges

Both HR and MAP increased during the mental and physical challenges, and to similar extents in the older adults with aMCI or normal cognition (Figure 2), suggesting similar activation of the cardiovascular responses in both groups (Wasmund et al., 2002). Although the two tests similarly increased MAP, HR increased more during the serial sevens test than isometric handgrip, suggesting that the mental arithmetic task elicited a greater cardiac response than isometric handgrip at 30% of the subject’s MVC. However, the increases in HR and MAP in healthy young subjects are remarkably greater during isometric handgrip test than mental arithmetic task (Wasmund et al., 2002). Interestingly, peak responses in HR (∆HR) and MAP (∆MAP) plateau within the 1st and 2nd min mental challenge, respectively, in young adults (Wasmund et al., 2002), similar to the temporal pattern of the present study (Figure 2). During mental challenge in these young subjects, however, ∆HR (+12 bpm) and ∆MAP (+8 mmHg) exceeded the respective responses in the elderly subjects of the present study. Moreover, both peak ∆HR and ∆MAP are significantly smaller during mental challenge vs. handgrip test in these young subjects (Wasmund et al., 2002), in contrast to the responses in the elderly subjects. Muscle contraction during handgrip elicits a pressor reflex (McCloskey and Mitchell, 1972) mediated by intramuscular metabo- and mechano-sensitive group III and IV afferents (Kaufman et al., 1983; Amann et al., 2010; Amann et al., 2011). Aging is associated with reduced effect of the intramuscular group III/IV afferents as evident by a diminished inhibitory influence of afferent pathway blockade on cardiovascular responses in older adults (Sidhu et al., 2015). It is likely that the smaller HR response to isometric handgrip vs. mental challenge could be related to a reduced pressor reflex in the elderly subjects with or without aMCI. Nonetheless, this augmented ∆HR in the aMCI and non-MCI subjects paralleled the enhanced ∆ScO_2_ and ∆O_2_Hb during the mental challenge and may have contributed to increased cerebral perfusion and tissue oxygenation during the mental challenge.

Interestingly, systemic arterial O_2_ saturation increased during serial sevens test in both the aMCI and non-MCI groups. This increased SaO_2_ could be ascribed to the mentally stressful serial sevens test and the attendant increased alveolar ventilation, which would increase arterial O_2_ content and, thus, SaO_2_; indeed, acute hyperventilation increases HR (Hawkins et al., 2019) and SaO_2_ (Lerant et al., 2015). The elevated SaO_2_ would contribute to enhanced tissue oxygenation during the mental challenge (Figure 3). In contrast, SaO_2_ tended to be decreased during physical challenge which might be associated with the isometric handgrip induced involuntary breathing holding.

### Impact of aMCI on cerebral oxygenation during mental and physical activity

Although mental stimulation provoked greater increases in HR, O_2_Hb and ScO_2_ than the physical challenge in elderly adults, none of these responses differed in the aMCI vs. non-MCI subjects. Furthermore, there were no significant differences in baseline HR, MAP, SaO_2_ and ScO_2_ between the two groups, although cerebral O_2_Hb was higher in the subjects with aMCI (Table 1). Henley et al. (2018) reported similar resting cardiovascular values between adults with or without MCI, but the cardiovascular responses to serial sevens and face-name recall tests were diminished in the MCI group. On the other hand, MRI-measured cerebral blood flow (CBF) at baseline is reportedly lower in MCI than non-MCI subjects (Chao et al., 2009; de Eulate et al., 2017; Kim et al., 2020; Liu et al., 2023). Since cerebral perfusion determines cerebral tissue oxygenation (Strangman et al., 2002), lower baseline CBF would predict reduced ScO_2_ and/or O_2_Hb in subjects with MCI. Enhanced baseline cerebral tissue oxygenation may help improve some aspects of cognitive function in aMCI subjects (Wang et al., 2020). However, in the present study baseline O_2_Hb was higher in the aMCI group (Table 2).

Liu et al. (2023) suggested that brain tissue loss could explain the reduced CBF in MCI subjects. By lowering cerebral oxygen consumption, either reduced brain mass or metabolism may decrease oxygen delivery requirements and, thereby, CBF. Thus, the higher baseline O_2_Hb in the aMCI subjects was likely ascribable to aMCI-associated reduced basal cerebrocortical oxygen consumption and neuronal activity and/or reduced brain tissue mass. Nevertheless, mental and physical tasks elicited similar increases in cerebrocortical oxygenation in the aMCI and non-MCI subjects.

### Study limitations and perspectives

This study employed isometric handgrip at 30% of MVC for 1 min as a moderately intense physical challenge intended to minimally impact the elderly subject’s neuromuscular, metabolic, and cardiovascular systems. Arguably, the more limited augmentation of HR during the 1-min physical challenge vs. 2-min arithmetic task might be related to the shorter duration of the former. However, the responses of ∆HR appeared to plateau within the first minute of the mental challenge (Figure 2), in agreement with the findings of Wasmund et al. in healthy young (ages 24–29 years) adults (Wasmund et al., 2002), where HR peaked in the 1^st^ min of isometric handgrip, in advance of increased muscle sympathetic activity.

Although prefrontal cortical ScO_2_ and O_2_Hb served as indicators of cerebral metabolism, cerebral blood flow (CBF) was not monitored during this study. CBF is a pivotal determinant of cerebrocortical oxygenation; thus, cerebral ScO_2_ and O_2_Hb correlated directly with CBF measured by functional MRI (Strangman et al., 2002) or transcranial Doppler sonography (Formes et al., 2010; Li et al., 2017; Liu et al., 2020) in human subjects. The more robust increases in cerebrocortical oxygenation during the mental vs. physical challenges provide neurophysiological support for potential applications of mental exercise training, which could especially benefit elderly subjects, in whom physical challenges impose increased cardiovascular demand (Takahashi et al., 2022) and particularly in those with MCI-associated reductions in CBF (Chao et al., 2009; de Eulate et al., 2017; Kim et al., 2020; Liu et al., 2023). Future studies with larger sample sizes are needed to compare the cerebral hemodynamic and oxygenation responses of aMCI subjects with simple vs. multiple short-term memory impairments. However, given the absence of statistically significant differences in cerebral oxygenation, HR or arterial pressure responses in the aMCI vs. non-MCI groups in this study, the degree or complexity of memory impairment among aMCI subjects may impact cerebrovascular function and cerebral oxygenation only modestly.

In summary, 2-min mental arithmetic elicited greater increases in heart rate than 1-min isometric handgrip, yet the two tasks elicited similar increases in mean arterial pressure, in elderly adults with normal cognition or aMCI. Cerebrocortical tissue oxygenation and oxygenated Hb increased during both tasks but to a greater extent during the mental challenge, supporting potential applications of mental exercises to improve cerebral blood flow and oxygen delivery in elderly adults. Although baseline cerebral oxyhemoglobin was higher in the elderly subjects with aMCI, none of these responses to mental or physical challenges were altered appreciably by the presence of aMCI. Thus, mental challenges to maintain brain health may be similarly effective in elderly adults with aMCI as in those with intact cognition.

## Data Availability

The raw data supporting the conclusions of this article will be made available by the authors, without undue reservation.
